# *Cyclocarya paliurus* Triterpenoids Improve Diabetes-Induced Hepatic Inflammation *via* the Rho-Kinase-Dependent Pathway

**DOI:** 10.3389/fphar.2019.00811

**Published:** 2019-07-25

**Authors:** Cuihua Jiang, Yiting Wang, Qiaomei Jin, Dongjian Zhang, Meng Gao, Nan Yao, Zhiqi Yin, Jian Zhang, Shiping Ma

**Affiliations:** ^1^Department of Pharmacology of Chinese Materia Medica, China Pharmaceutical University, Nanjing, China; ^2^Affiliated Hospital of Integrated Traditional Chinese and Western Medicine, Nanjing University of Chinese Medicine, Nanjing, China; ^3^Jiangsu Province Academy of Traditional Chinese Medicine, Nanjing, China

**Keywords:** *Cyclocarya paliurus*, triterpene, diabetes mellitus, hepatic inflammation, ROCK, NF-κB

## Abstract

This study aimed to assess the effects of triterpene extract of *Cyclocarya paliurus* (Batal.) Iljinskaja (CPT) on diabetes-induced hepatic inflammation and to unveil the underlying mechanisms. Diabetes in db/db mice was alleviated after CPT administration, as assessed by the oral glucose tolerance test. In addition, treatment with CPT dramatically reduced serum insulin, aspartate amino-transaminase, alanine aminotransferase, triglyceride, and total cholesterol amounts. Besides, serum levels of interleukin (IL)-1β, IL-6, and tumor necrosis factor (TNF)-α were also reduced after CPT administration. Western blot analysis revealed that CPT treatment significantly reversed the protein expression levels of Rho, ROCK1, ROCK2, p-P65, p-IκBα, p-IKKα, and p-IKKβ in liver samples obtained from db/db mice. Upon palmitic acid stimulation, the protective effects of CPT on the liver were further assessed in HepG2 and LO2 cells, and no appreciable cytotoxic effects were found. Therefore, these findings indicate that CPT alleviates liver inflammation *via* Rho-kinase signaling.

**Chemical compounds evaluated in this report:** Metformin (PubChem CID: 4091); Fasudil (PubChem CID: 3547); Palmitic acid (PubChem CID: 985).

## Introduction

Diabetes mellitus (DM), a chronic ailment associated with metabolic disorders, features chronic hyperglycemia caused by insulin deficiency or resistance and is considered a serious global health burden (Makimattila et al., 1996; Abajobir et al., 2017). It was reported that an estimated 451 million people suffered from diabetes between the ages of 18 and 99 years in 2017 worldwide, and these figures are anticipated to increase to 693 million by 2045 (Cho et al., 2018). DM damages multiple organs to elicit severe complications such as blindness, heart attack, gangrene, and liver cirrhosis, and these multiple abnormalities impose enormous clinical and economic burden on patients, families, and society (Harrison, 2006; Van Dieren et al., 2010; Seuring et al., 2015). A growing body of evidence suggests that chronic hyperglycemia affects inflammatory mechanisms in liver, and it in turn drives hepatic injury in the aspects of lipid accumulation and liver fibrosis (Kelley et al., 2003; Hui et al., 2004; Polyzos et al., 2011; Chi et al., 2012; Dharmalingam and Yamas, 2018). Thus, hepatic inflammation represents a causative mechanism among these responses. Furthermore, some anti-inflammatory agents have important functions in increasing insulin sensitivity, ameliorating glucose control and attenuating live injury in several studies (Renata et al., 2006; Ford et al., 2015; MB et al., 2016). Therefore, therapeutic inhibition of hepatic inflammation may be potential strategies for DM and metabolic abnormalities (Saremi et al., 2010).

Rho-associated coiled-coil-containing protein kinase (ROCK), a serine-/threonine-protein kinase with two isoforms ROCK1 and ROCK2, is the downstream effector molecule of Rho which belongs to the Ras superfamily of small monomeric GTPase. Indeed, Rho-related kinases constitute an array of pathological and physiological signals induced in diabetes and are considered potential targets for nephroprotective treatments in diabetes (Komers, 2013; Mishra et al., 2013; Nandipati et al., 2017). Clinical evidence showed that ROCK activity is significantly increased in type 2 diabetic patients (Liu et al., 2016). Furthermore, recent studies confirmed that Rho/ROCK pathway activation is observed in multiple diabetic complication such as cardiovascular diseases, renal disease, neuropathy, and hepatic fibrosis (Peng et al., 2008; Nunes et al., 2010; Mishra et al., 2013; Zhou et al., 2014). In addition, ROCK activation can upregulate expression of tumor necrosis factor (TNF-α) and NF-κB (Segain et al., 2003; Xie et al., 2016b), both of which impact macrophage infiltration to tissue and ectopic lipid accumulation. Fasudil as the first-generation selective Rho/ROCK inhibitor ameliorated the activation of ROCK and NF-κB in the livers of diabetic rats, exerting a beneficial adjuvant drug for diabetes and its complications (Zhou et al., 2014; Xie et al., 2018). In short, these results indicate the therapeutic promising of the Rho/ROCK pathway as a novel target against diabetes (Jahani et al., 2018).

*Cyclocarya paliurus* (Batal.) Iljinskaja *(C. paliurus)* (CP), a traditional Chinese medicinal herb, is widely used as daily beverage for obesity and diabetes prevention or treatment. It is authorized as new food raw material in 2013 by National Health and Family Planning Commissions of the People’s Republic of China (Xie et al., 2016a). Our previous researches revealed that CP leaf extracts featured pharmacological activities such as anti-inflammation, anti-hyperglycemia, and anti-hyperlipidemia activities and revealed that triterpenoids were its primary active compound (Jiang et al., 2014; Zhu et al., 2015; Wu et al., 2017; Zhao et al., 2018). However, definitely investigating the molecular mechanisms how the CP combats diabetes is still deficient. Triterpenoids are attractive targets for phytomedicinal research because of multiple strong pharmacologic and biological activities. Oleanolic acid and betulinic acid not only display cytotoxic activity toward cancer cells but also effectively ameliorates intracellular lipid accumulation in liver (Quan et al., 2013; Somwong and Suttisri, 2018). Arjunolic acid can inhibit the excessive ROS and RNS formation as well as reduce steatosis and MNC infiltration, promising lead to treat diabetes and NAFLD (Manna et al., 2009; Toppo et al., 2018). Moreover, celastrol and ursolic acid attenuate mesangial cell injury and decrease lipid synthesis, which improve metabolic damage (Lu et al., 2015). Therefore, this work aimed to assess the hepatoprotective properties of triterpenoid-enriched fraction from CP by analyzing diabetes-induced hepatic inflammation and exploring Rho-kinase signaling underlying such effects.

## Materials and Methods

### Materials and Reagents

Fasudil (fasudil hydrochloride injection) was obtained from Tianjin Chase Sun Pharmaceutical. Co., Ltd. (Tianjin, China). Metformin was manufactured by Sino-American Shanghai Squibb Pharmaceuticals Ltd. (Shanghai, China). Enzyme-linked immunosorbent assay (ELISA) kits for measurement of IL-1β, IL-6, and TNF-α were manufactured by Nanjing KeyGEN Biotech. Co., Ltd. (China). Specific ELISA kit to determine lactic dehydrogenase (LDH) was purchased from Bioswamp (Wuhan, China). Commercial kits to determine total cholesterol (TC), triglyceride (TG), aspartate amino-transaminase (AST), and alanine aminotransferase (ALT) were provided by Nanjing Jiancheng Bioengineering Institute (Nanjing, China). Antibodies were purchased from Cell Signaling Technology (Boston, USA).

### Drug Preparation and Analysis

*C. paliurus* leaves provided from Nanjing Forestry University (GPS coordinates, 118.822414, 32.085054) underwent authentication by Prof. Min-Jian Qin (China Pharmaceutical University, China) and recorded with a voucher specimen (No. L20100033) at the Department of Natural Medicinal Chemistry of our university. The CPT sample was prepared as reported in a previous study (Wu et al., 2017). Briefly, pulverized air-dried CP leaves (1.5 kg) were extracted three times with 12 L of 80% ethanol under reflux for 2 h. After combining the three extracts, the resulting samples was defatted (petroleum ether) and partitioned with chloroform three times to afford a chloroform fraction. The latter fraction was separated using 3% NaOH in water followed by neutralization with 5% HCl. Subsequently, three re-extractions were performed using chloroform to provide the triterpene-enriched extract (CPT, 50.8 g). The triterpene-enriched fraction was dissolved in HPLC grade methanol to prepare sample solution for HPLC analysis as Wu reported (Dongsheng et al., 2005). The solvent system composed of 0.01% formic acid water (A) and 0.01% formic acid-acetonitrile (B) in the following gradient: 0–13 min, 8–19% B; 13–28 min, 19–21% B; 28–42 min, 21–50% B; 42–46 min, 50% B; 46–60 min, 50–55% B; 60–64 min, 55–56% B; 64–74 min, 56–66% B; 74–94 min, 66–85% B; 94–114 min, 85–95% B; 114–119 min, 95–100% B; 119–125 min, 100% B; 125–140 min, 100% B–8% B; and 140–150 min, 8% B. Operating conditions were as follows: detection wavelength, 205 nm; flow rate, 1.0 ml/min; column temperature, 45°C; and injection volume, 10 μl.

### Animal Studies

Male db/db and wild type mice (8 weeks old) on the C57BL/KsJ background were provided by Model Animal Research Center of Nanjing University (China). The animals had freely available food and water and were housed under 12 h–12 h light/dark cycles at 23 ± 2°C. Six animal groups were set up with mice randomly assigned: wild type, db/db, metformin (20 mg/kg), CPT-L (0.25 g/kg), CPT-H (0.5 g/kg), and fasudil (5 mg/kg) groups. After 1 week of adaptive feeding, mice were administrated drugs daily for 4 weeks and euthanized by an overdose of anesthetics. Body weight was measured every week. Then, serum specimens and livers were obtained for further studies. All animal experimental procedures had approval from China Pharmaceutical University (CPU.2012-003) and were performed according to the National Institutes of Health Guidelines for the Care and Use of Laboratory Animals (NIH Publications No. 8023, revised 1978).

### Oral Glucose Tolerance Test

For the oral glucose tolerance test (OGTT), mice received an oral administration of 2 g/kg glucose at 8:00 am after overnight fast. Tail vein blood specimens were obtained from mice at 0, 30, 60, 90, and 120 min, respectively, upon glucose administration. Blood glucose was detected with a Surestep glucose analyzer (LifeScan, Inc., CA).

### Histological Analysis

Livers were collected from euthanized animals and submitted to fixation with 10% formalin. After paraffin embedding, 3-μm sections were obtained and submitted to hematoxylin–eosin (H&E) staining as directed by the manufacturer. Liver sections were immunostained with rabbit anti-insulin polyclonal antibodies (Cell Signaling, Danvers, MA) followed by incubation with the avidin–biotin peroxidase complex (DAKO, CA). Stained sections were imaged to observe pathological changes under a light microscope.

### Cell Culture and Treatment

HepG2 and LO2 cells from Shanghai Cell Bank of the Chinese Academy of Sciences were cultured in Dulbecco’s modified eagle’s medium (DMEM) containing 10% fetal bovine serum (FBS) and 1% double antibiotics (penicillin and streptomycin) in a humid environment with 5% CO_2_ at 37°C. Cells were seeded into six-well plates until a confluency between 70% and 80%. The cells then were pretreated with CPT (2, 4, and 8 μg/ml) and fasudil (10 μM) for 2 h prior to incubation with or without palmitic acid (100 μM) for 6 h under serum-starvation conditions.

### Cytotoxicity Assay

Live cells were quantitated by the MTT assay. HepG2 and LO2 cells were plated in 96-well plates (5 × 10^3^/well), incubated with different CPT (1, 2, 4, 8, and 16 μg/ml) amounts for 2 h, and treated with palmitic acid for 6 h prior to incubation with 5 mg/ml MTT (Sigma, Cat. #M2128) for 4 h. After incubation, the solution was discarded, and formazan crystals were solubilized with 150 μl/well dimethyl sulfoxide (Cat. #75927N). Optical density was detected at 570 nm on a microplate spectrophotometer. Cell viability (%) was determined as (A_Treated_/A_Control_) × 100%. The aim of LDH release assay was to determine the quantification of cytotoxicity based on the release of cytoplasmic LDH into culture media from damaged cells. HepG2 and LO2 cells were treated as above, and cell supernatant was collected. LDH release into media was measured according to the manufacturer’s protocol.

### Analysis of Serum Samples and Cell Supernatants

TC, TG, AST, ALT, and insulin amounts in mouse serum specimens and HepG2 cell supernatants after treatment with palmitic acid were assessed by ELISA. In addition, IL-6, IL-1β, and TNF-α amounts in serum samples, liver tissue specimens, and HepG2 cell supernatants were evaluated with respective ELISA kits, as directed by the manufacturer. These cytokines were quantified using standard curves. All other chemicals used were of analytical grade.

### Immunoblot

Liver tissue specimens and HepG2 cells underwent lysis with the RIPA (Radio-Immunoprecipitation assay) lysis buffer (CST, Cat. #9806). Total protein was extracted by centrifugation at 12,000 rpm for 20 min and quantified by the BCA (Bicinchoninic acid) protein assay method (Beyotime). Equal amounts of total protein were resolved by 10% SDS-polyacrylamide gel electrophoresis (SDS-PAGE) and electro-transferred onto polyvinylidene fluoride (PVDF) membranes. After blocking in 5% nonfat milk 2 h at room temperature, the membranes were incubated with the following antibodies overnight at 4°C: anti-Rho, anti-ROCK1, anti-ROCK2, anti-p-P65, anti-P65, anti-p-IκBα, anti-IκBα, anti-p-IKKα, anti-IKKα, anti-p-IKKβ, anti-IKKβ, and anti-GAPDH. Then, three TBST washes were performed, followed by incubation with horseradish peroxidase (HRP)-linked secondary antibodies for 2 h in ambient conditions. The enhanced chemiluminescence method was employed to detect immunoreactive bands using a gel imaging system (Tanon Science & Technology Co., Ltd., China).

### Immunohistochemical Analysis

Livers were removed from euthanized animals at the end of experiment and post-fixed in 4% PFA at 4°C overnight. Then, 5-μm sections were obtained, submitted to permeabilization for 2 h with 0.4% Triton X-100 (Santa Cruz, Cat. #sc-29112) and blocking (1% BSA for 2h in ambient conditions). Each section was next sequentially stained with primary (72 h at 4°C) and secondary (2 h in ambient conditions) antibodies. After PBS washes, the slides were dehydrated, followed by mounting with the VECTASHIELD mounting medium. Imaging was performed under a Leica confocal microscope.

### Statistical Analysis

All values represent three or more separate experiments and are mean±SD. Multiple experimental groups were assessed by one-way analysis of variance (ANOVA) followed by the Tukey multiple comparison test. *P* < 0.05 indicated statistical significance.

## Results

### Phytochemical Analysis

**Figure 1:**
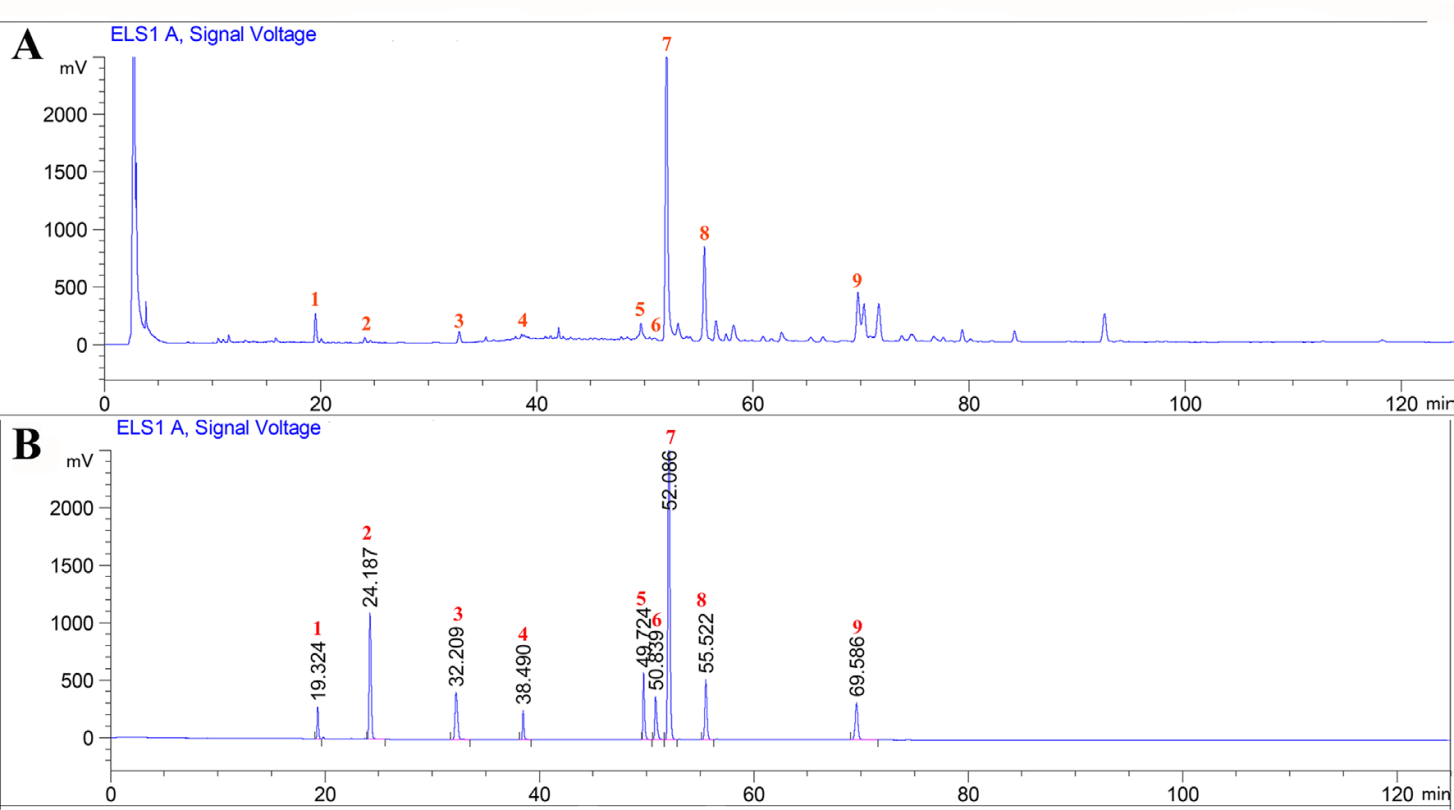
High-performance liquid chromatography profiles of *Cyclocarya paliurus* triterpenoids fraction **(A)** as well as standard mixture **(B)** using ELSD (Evaporative light Scattering Detector) detection. External standards: 1) isoquercitrin, 2) kaempferol-3-O-β-D-glucoside, 3) kaempferol-3-O-α-L-rhamnopyranoside, 4) quercetin, 5) 2a,3β,23-trihydroxyoleana-12, 20(30)-dien-actinidic acid, 6) 2a,3β,23-trihydroxyoleana-11,13(18)-dien-28-oic acid, 7) arjunolic acid, 8) cyclocaric acid B, and 9) 3β,23-dihydroxy-12-ene-28-ursolic acid.

The major chemical components of CPT were revealed by HPLC analysis as shown in **Figure 1**. A comparison with corresponding reference standards revealed the presence and relative content of individual constituent in CPT fraction as follows: 1) isoquercitrin (46.75±0.25 mg/g), 2) kaempferol-3-O-β-D-glucoside (32.66±1.07 mg/g), 3) kaempferol-3-O-α-L-rhamnopyranoside (25.24±0.70 mg/g), 4) quercetin (41.98±1.32 mg/g), 5) 2a,3β,23-trihydroxyoleana-12,20(30)-dien-actinidic acid (11.76±0.39 mg/g), 6) 2a,3β,23-trihydroxyoleana-11,13(18)-dien-28-oic acid (50.46±0.62 mg/g), 7) arjunolic acid (214.90±1.96 mg/g), 8) cyclocaric acid B (112.53±1.72 mg/g), and 9) 3β,23-dihydroxy-12-ene-28-ursolic acid (27.58±1.86 mg/g).

### Effect of CPT on OGTT and Body weight

**Figure 2:**
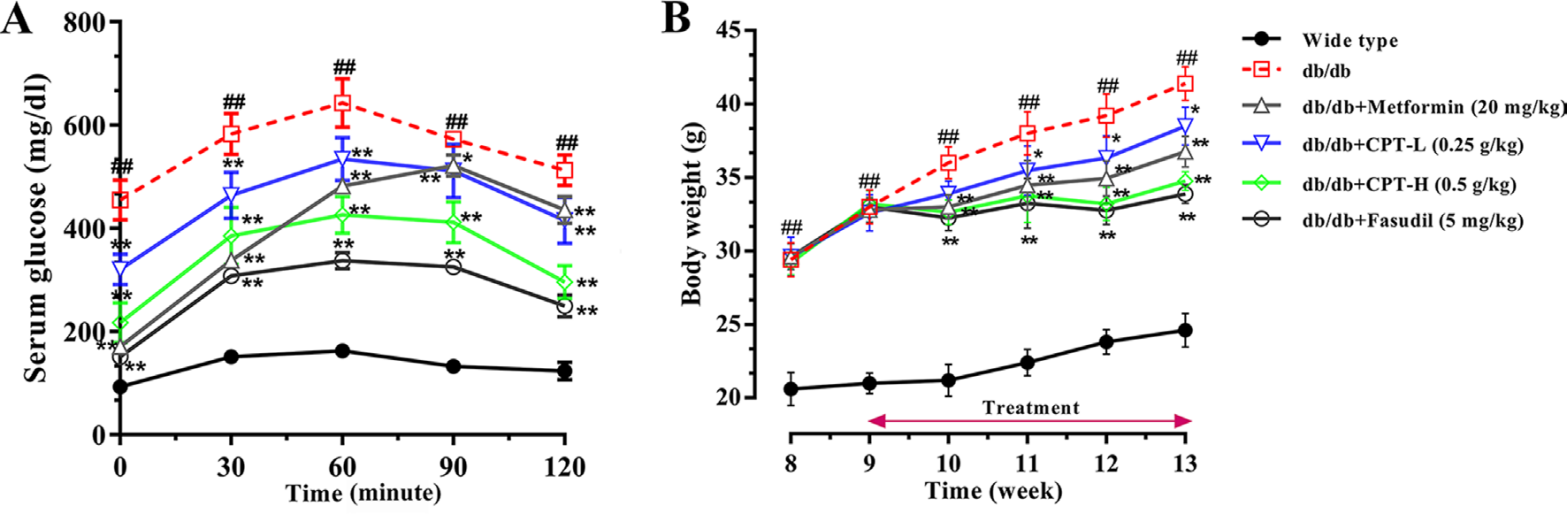
CPT’s impact on OGTT **(A)** and body weight **(B)**. Data are mean ± SD. ^##^ *p* < 0.01, ^#^ *p* < 0.05 *vs*. wild type animals. **p < 0.01, *p < 0.05 *vs*. db/db mice.

As shown in **Figure 2A**, glucose exposure (per os) significantly increased blood glucose levels in db/db mice group as compared with wide-type group, and a gradual reduction was present after CPT (0.25 or 0.5 g/kg) treatment. Blood glucose levels in db/db animals administered with CPT (0.25 or 0.5 g/kg) showed a significant increase at 30 min, but reversed to basal amounts within 2 h of glucose ingestion; a comparable trend was observed for the metformin and fasudil groups. Meanwhile, CPT significantly reduced body weight gain of mice in db/db mice at the second week after CPT administration in comparison with db/db group, which was defined at the fourth week as shown in **Figure 2B**. A comparable effect was shown in metformin and fasudil groups.

### CPT Protects the Liver in Diabetic Mice

**Figure 3:**
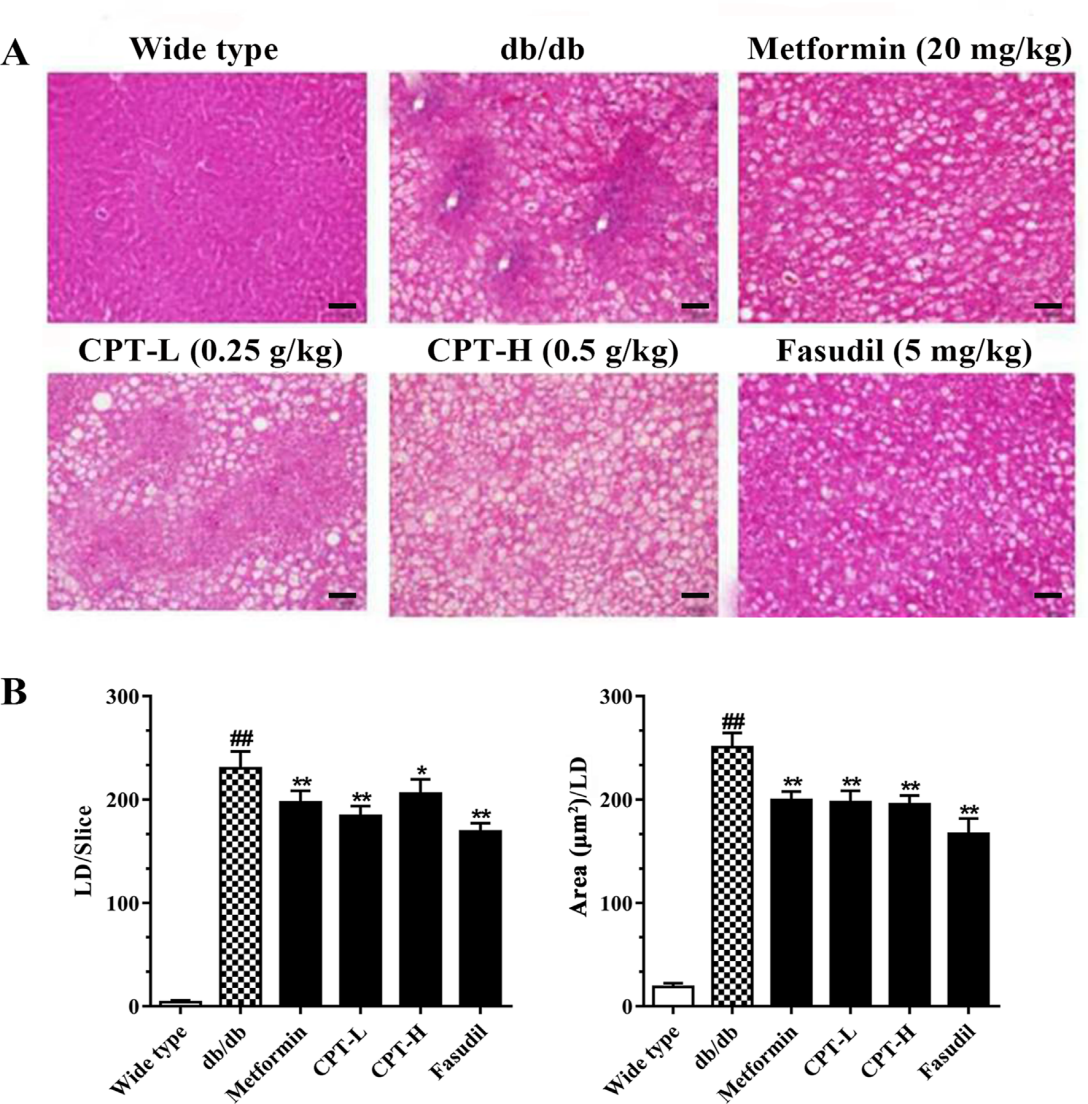
CPT’s effects on histological features **(A)** as well as the size and number of lipid droplets **(B)**. Data are mean ± SD. ^##^ *p* < 0.01, ^#^ *p* < 0.05 *vs*. wild type animals. **p < 0.01, *p < 0.05 *vs*. db/db mice. Scale bars = 100 µm.

Histological assessment revealed macrovesicular steatosis in hepatic tissue samples from the db/db group (**Figure 3**). Meanwhile, CPT, metformin, or fasudil decreased the extent of steatosis and remarkably reduced lipid droplets (size and number) in diabetic animals, indicating that these treatments significantly inhibited ectopic lipid accumulation. The above findings revealed that CPT effectively improved the hepatocyte ballooning, inflammatory cell infiltration, and hepatic steatosis in db/db mice.

### CPT Increases Liver Cell Viability

**Figure 4:**
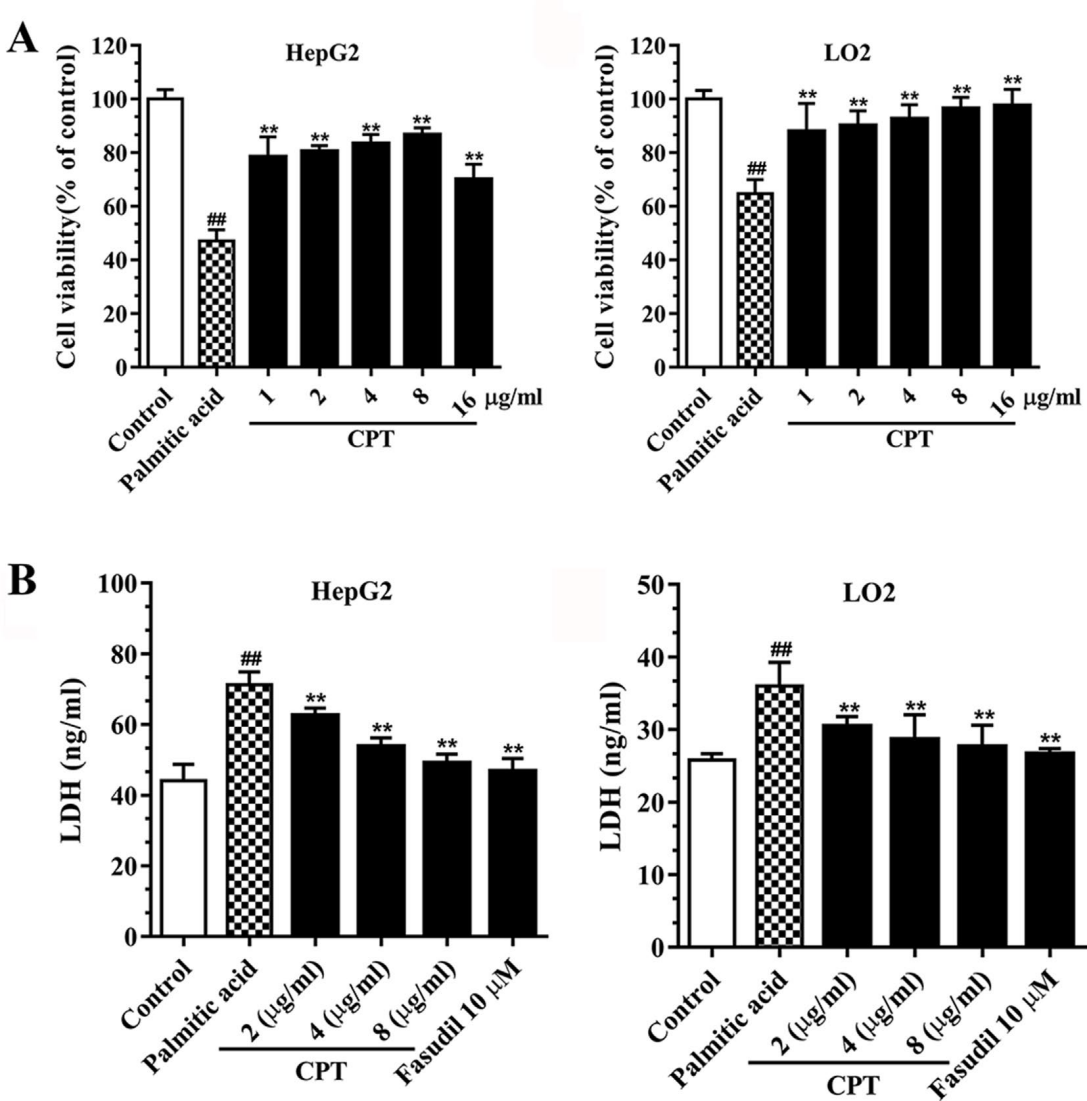
CPT’s effects on cell viability **(A)** and LDH release **(B)** in HepG2 and LO2 cells. Data are mean ± SD. ^##^ *p* < 0.01, ^#^ *p* < 0.05 *vs*. control cells. **p < 0.01, *p < 0.05 *vs*. palmitic acid group.

Palmitic acid inhibited HepG2 and LO2 cell viabilities, while CPT (2, 4, 8 μg/ml) and fasudil (10 μM) efficiently enhanced HepG2 and LO2 cell viability after treatment with palmitic acid. Besides, CPT (2, 4, 8 μg/ml) and fasudil (10 μM) significantly reduced palmitic acid–induced LDH release of HepG2 and LO2 cells. This suggested that CPT could alleviate palmitic acid induced cell damage and increase cell viability (**Figure 4**).

### Effects of CPT on Serum and Secreted Biomarkers

**Figure 5:**
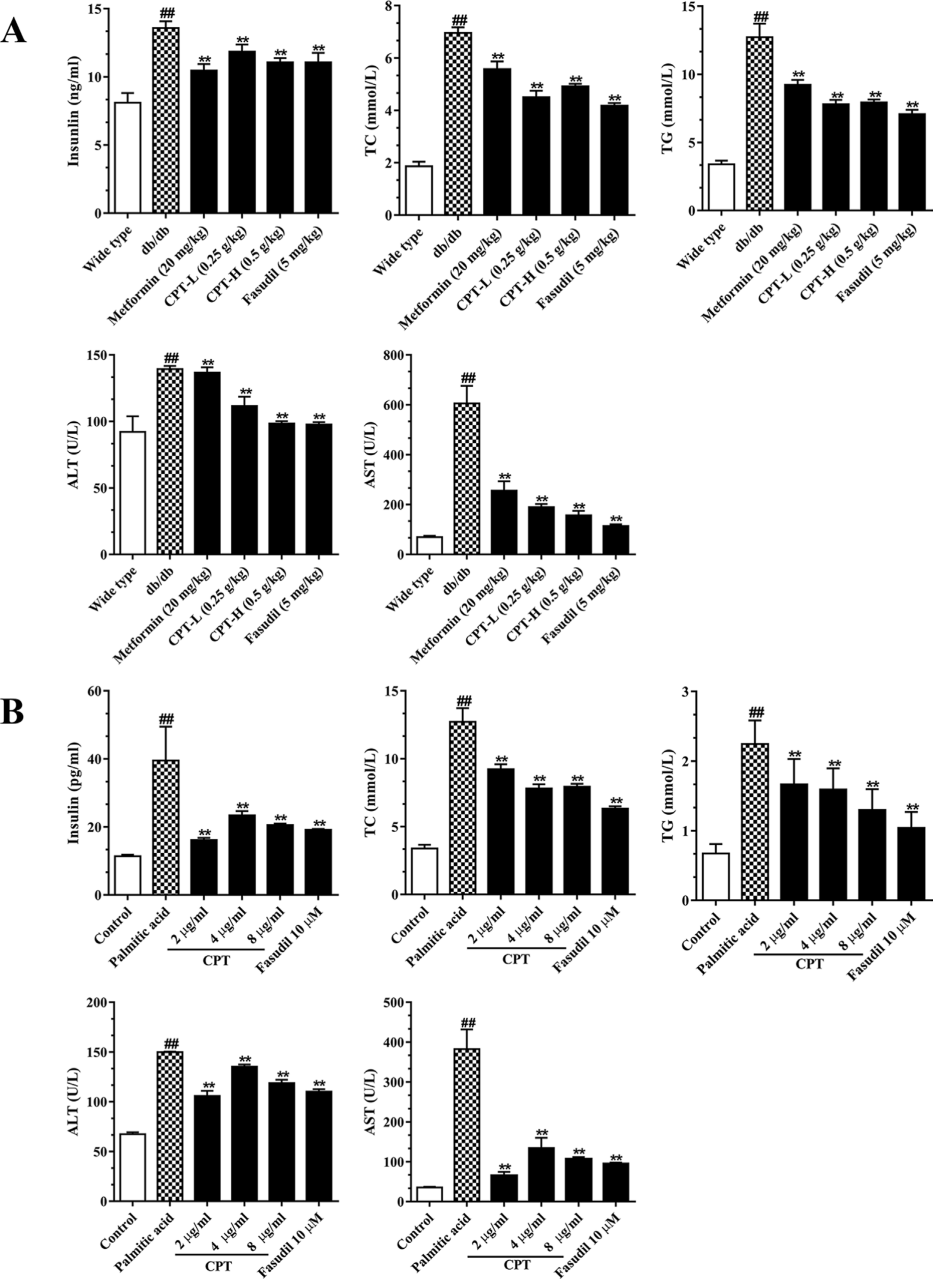
CPT’s on serum **(A)** and cell supernatant **(B)** levels of various biomarkers. **(A)** Data are mean ± SD. ^##^ *p* < 0.01, ^#^ *p* < 0.05 *vs*. wild type mice. **p < 0.01, *p < 0.05 *vs*. db/db mice. **(B)** Data are mean ± SD. ^##^ *p* < 0.01, ^#^ *p* < 0.05 *vs*. control cells. **p < 0.01, *p < 0.05 *vs*. palmitic acid group.

Serum TC, TG, insulin, AST, and ALT amounts were significantly increased in the db/db group in comparison with wild type animals (**Figure 5A**). However, CPT, metformin, and fasudil, respectively, reversed the above effects. After palmitic acid challenge, the levels of TC, TG, insulin, ALT, and AST were increased in HepG2 cells; meanwhile, CPT pretreatment successfully reduced their amounts, with the 2 μg/ml dose showing more pronounced effects (**Figure 5B**). Thus, CPT effectively ameliorated the “lipotoxic state” and hepatic IR in db/db mice, which demonstrated that CPT prominently prevented the progression to hepatic fibrosis.

### CPT Reduces the Amounts of Inflammatory Cytokines in Serum Samples and Cell Supernatants

**Figure 6:**
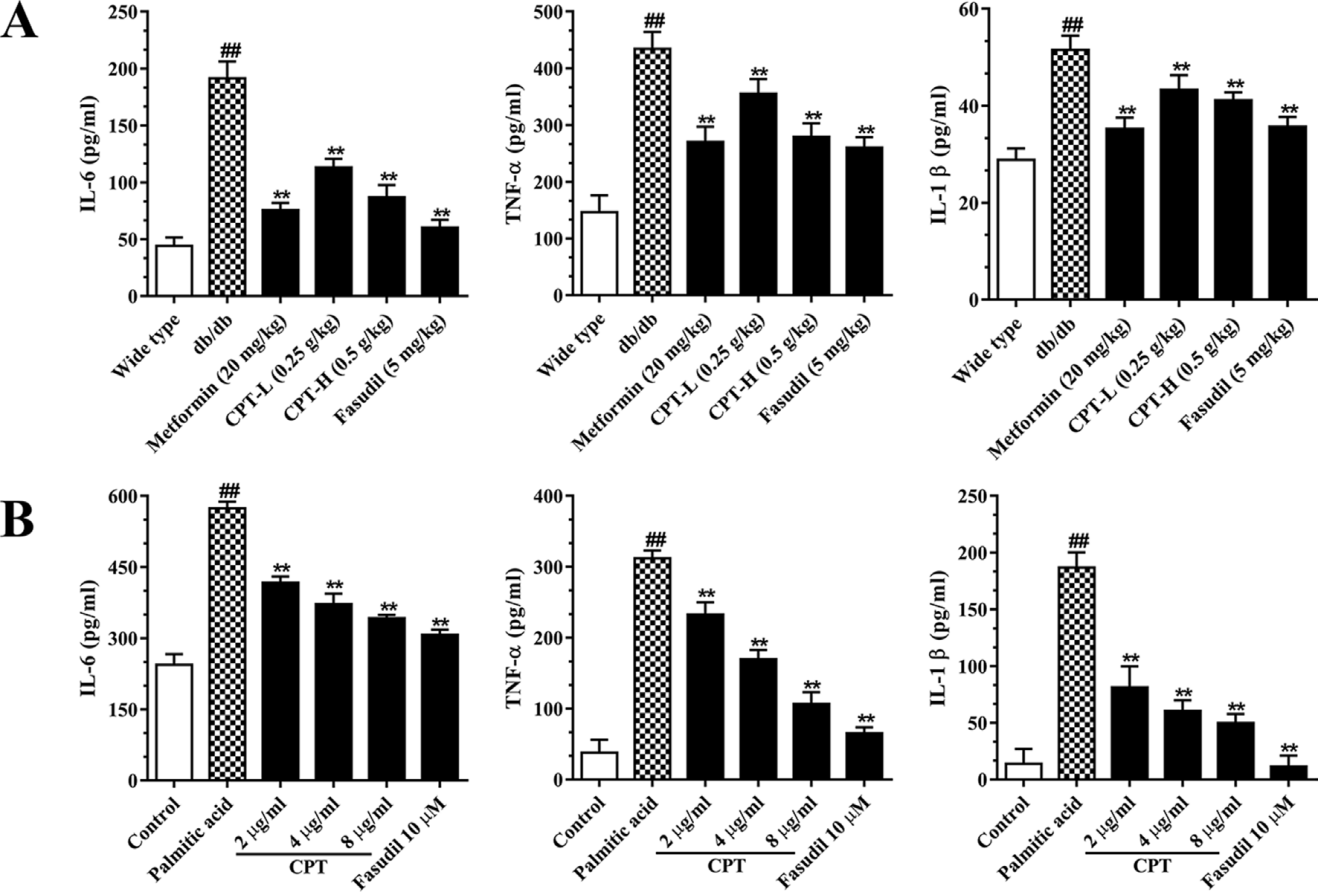
CPT’s effects on serum **(A)** and cell supernatant **(B)** levels of inflammatory cytokines. **(A)** Data are mean ± SD. ^##^ *p* < 0.01, ^#^ *P* < 0.05 *vs*. wild type mice. **p < 0.01, *P < 0.05 *vs*. db/db mice. **(B)** Data are mean ± SD. ^##^ *p* < 0.01, ^#^ *P* < 0.05 *vs*. control cells. **p < 0.01, *p < 0.05 *vs*. palmitic acid group.

Hepatic inflammation represents an important feature of liver damage associated with diabetes as mentioned above. Increased pro-inflammatory in DM adversely accentuated hepatic/systemic IR and increased the risk of HCC (Plessis et al., 2015). Metformin and pioglitazone, both of which are the fist-line therapeutic agent in the treatment of DM with hepatic damage, histologically ameliorate hepatic steatosis or inflammation as well as improved survival in cirrhosis and HCC (White et al., 2015; Somwong and Suttisri, 2018). To assess whether CPT improves inflammatory responses in diabetes-induced hepatic inflammation, we assessed IL-6, IL-1β, and TNF-α amounts in serum (**Figure 6A**). The results revealed markedly increased serum amounts of these biomarkers in the db/db group. On the contrary, the CPT, metformin, and fasudil treatment groups, respectively, had significantly decreased serum amounts of IL-6, IL-1β, and TNF-α in comparison with model mice. In line with these results, palmitic acid challenged HepG2 cells secreted overtly increased amounts of IL-6, IL-1β, and TNF-α in comparison with control cells; CPT and fasudil effectively reversed these effects (**Figure 6B**).

### Effects of CPT on Rho-Kinase Pathway

**Figure 7:**
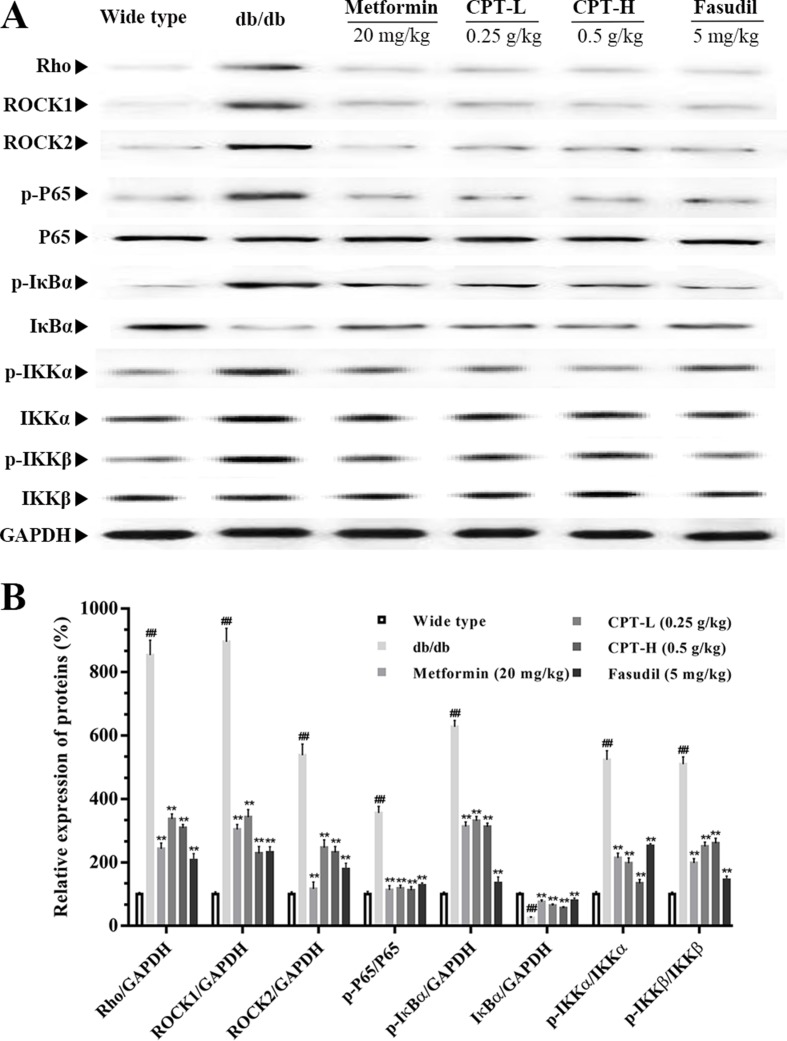
CPTs on the Rho-kinase pathway in mice. The protein levels of RHO and NF-κB signaling pathway in db/db mice treated with CPT, Metformin and Fasudil **(A, B)**. Data are mean ± SD. ^##^ *p* < 0.01, ^#^ *p* < 0.05 *vs*. wild type mice. **p < 0.01, *p < 0.05 *vs*. db/db mice.

**Figure 8:**
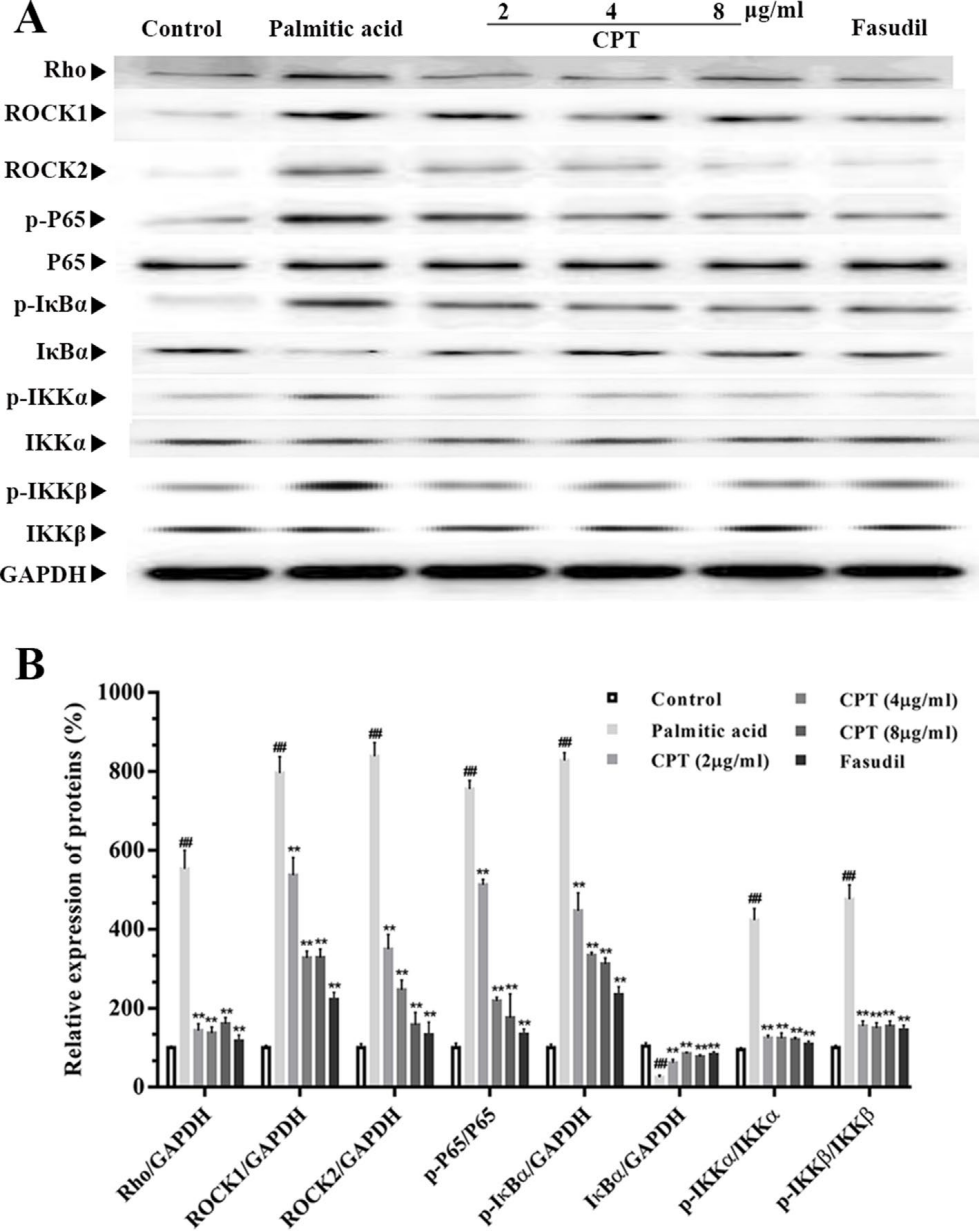
CPT’s effects on the Rho-kinase pathway in palmitic acid–treated HepG2 cells. The protein levels of RHO and NF-κB signaling pathway in palmatic acid-induced HepG2 cells treated with CPT, Metformin and Fasudil **(A, B)**. Data are mean ± SD. ^##^ *p* < 0.01, ^#^ *P* < 0.05 *vs*. control cells. **p < 0.01, *p < 0.05 *vs*. palmitic acid group.

Rho-kinase signaling plays an important role in the regulation of inflammatory cascades (**Figures 7** and **8**). The results indicated that protein expression levels of Rho, ROCK1, and ROCK2 were notably increased in liver samples from db/db mice. However, administration of CPT, metformin, and fasudil, respectively, obviously attenuated these effects. To clarify downstream mechanisms underlying CPT effects on diabetes-associated hepatic inflammation in db/db animals, IκB, NF-κB, IKKα, and IKKβ phosphorylation levels were assessed. We found that phosphorylation of these proteins was enhanced in the db/db group in comparison with wild type controls. Meanwhile, CPT (0.25 or 0.5 g/kg) overtly suppressed diabetes-associated phosphorylation of the above molecules. Meanwhile, metformin (20 mg/kg) and fasudil (5 mg/kg), respectively, also exerted comparable suppressive effects on the NF-κB signaling pathway. Similar trends were observed *in vitro*, with elevated Rho and NF-κB signaling activities after palmitic acid administration in HepG2 and LO2 cells (**Supplementary Figure S1**) in comparison with control cells. However, CPT and fasudil effectively reversed the above effects. Therefore, these findings proved that ROCK1 inhibition was capable of preventing NF-κB activation.

### Immunohistochemical Analysis

**Figure 9:**
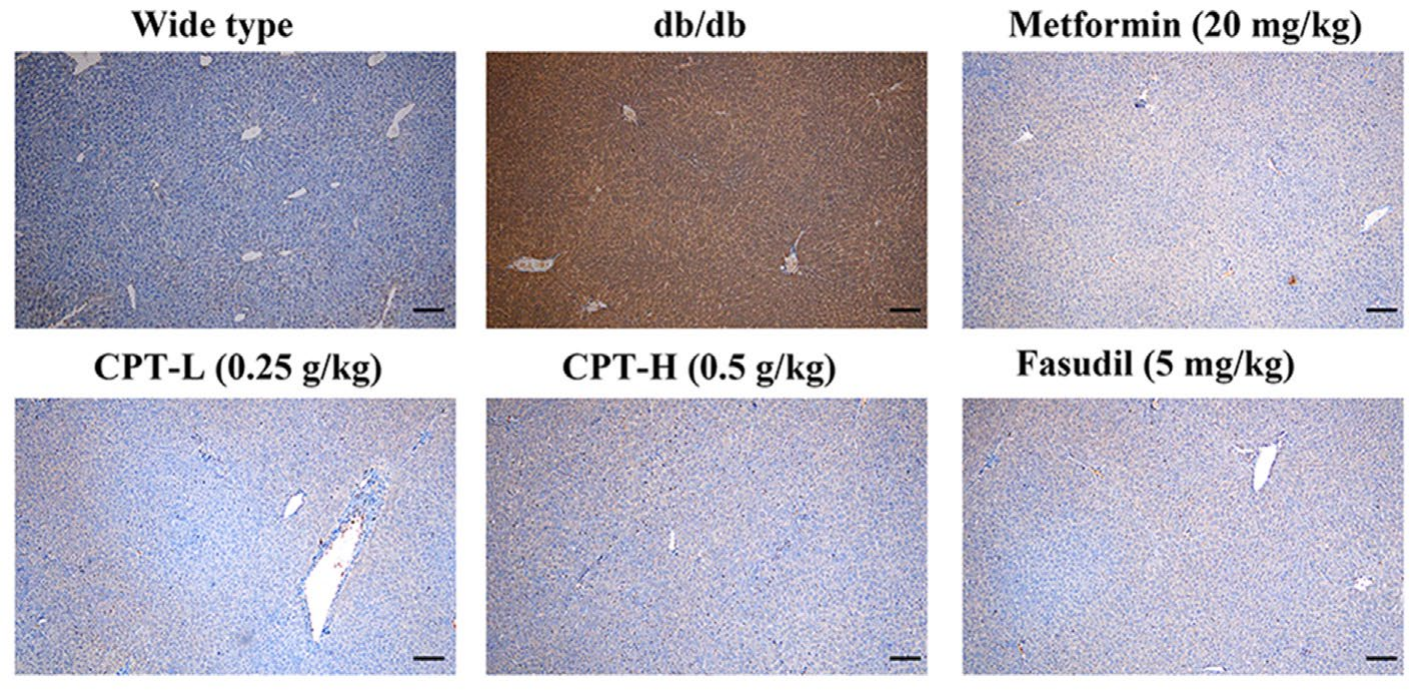
CPT’s impact on Rho protein levels in hepatic tissue specimens from db/db mice. Scale bars = 200 µm.

**Figure 10:**
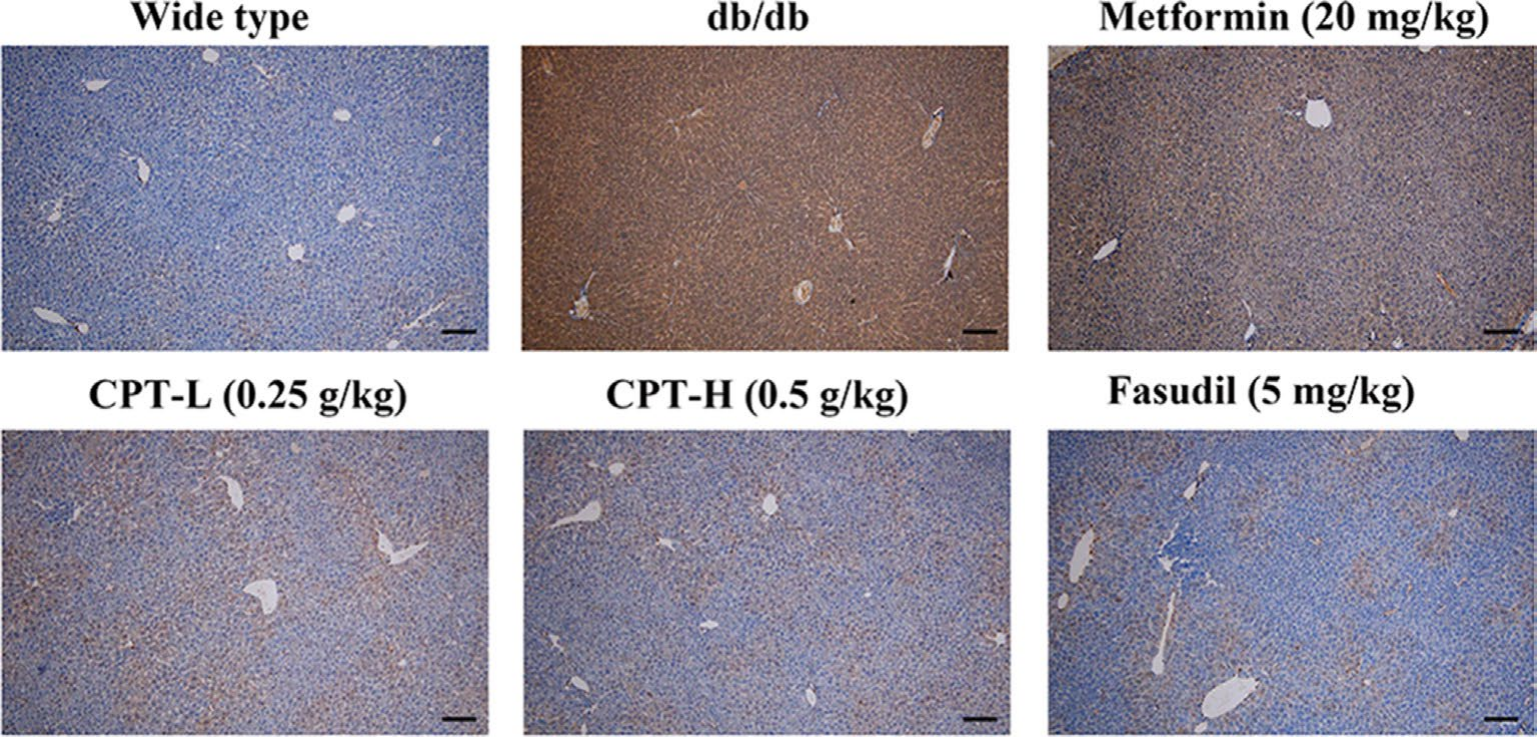
CPT’s impact on p-P65 protein levels in hepatic tissue specimens from db/db mice. Scale bars = 200 µm.

Rho-kinase signaling is critical in regulating inflammatory cascades. As shown in **Figures 9** and **10**, Rho and p-P65 amounts were markedly increased in hepatic tissue specimens from db/db mice; however, administration of CPT (0.25 or 0.5 g/kg), metformin (20 mg/kg), or fasudil (5 mg/kg) overtly ameliorated the above effects.

## Discussion

Diabetes is mainly characterized by hyperglycemia, lack of insulin activity and insulin, and is one of the fastest growing health issues across the world. The emerging pandemic is driven by rising levels of obesity, ageing, and longevity. Stress hyperglycemia exacerbates diabetic pathologies in the retina, kidney, nerve, liver, brain, and lower limbs through multiple abnormalities arising (Alberti et al., 1998; Alexander et al., 2000; Van Dieren et al., 2010). Chronic inflammation represents critical factor in the development of diabetes in metabolically active tissues such as the liver. In this study, we found that CPT effectively inhibited ectopic fat accumulation in diabetic mice liver. Furthermore, CPT significantly ameliorated OGTT as well as TC, TG, insulin, ALT, and AST amounts in diabetic mice and palmitic acid administered HepG2 cells. CPT not only significantly improved lipid metabolism and insulin responsiveness but also decreased circulating cytokines such as IL-6 and TNF-a. Moreover, CPT also reduced the hepatic expression of Rho-kinase and NF-κB, both of which are suggested to be a key link between hepatic inflammation and insulin resistance *in vivo* and *in vitro*. Taken together, this study demonstrated the beneficial effects of CPT on diabetic-induced hepatic inflammation.

Epidemiological findings indicating diabetes is a risk factor for liver damages such as HCC (El-Serag et al., 2004; Dharmalingam and Yamas, 2018). During type 2 diabetes (T2DM) progression, chronic inflammation represents a critical mechanism behind the pathogenetic process of liver damages (Kim et al., 2009). Increased FFA in diabetes flux into the ectopic tissues due to an increased rate of lipolysis aggravates hepatic IR, and the “lipotoxic state” results in hepatocyte necroinflammation (Plessis et al., 2015). In addition, hyperglycemia leads to transcriptional alterations and biochemical changes; then, many of these changes are attributed to oxidative stress. Increased presence of oxygen radicals further stimulate cytokine production and extracellular matrix proteins and the second “hit” that causes an increase in fibrosis (Giacco and Brownlee, 2010). Our data showed that CPT remarkably reduced TC, TG, insulin, ALT, and AST amounts in the “lipotoxic state.” Moreover, CPT significantly decreased content of TNF-a, IL-6, and IL-1β caused by second “hit” in diabetes. Thus, CPT not only significantly ameliorated lipid metabolism and insulin responsiveness but also decreased hepatic inflammation in diabetes.

Rho kinase (ROCK) can be divided into two isoforms: ROCK1 and ROCK2. ROCK is activated by RhoA, a member of the small G-protein group of the Rho family (Chen et al., 2015). ROCK signaling is associated with multiple ailments such as high blood pressure and other cardiovascular diseases. Indeed, Rho-related kinases constitute an array of pathological and physiological signals induced by diabetes and are considered potential targets for diabetes management (Komers, 2013). As previously reported, induced ROCK signaling is found in db/db mice, but selectively blunted by its competitive inhibitors (MB et al., 2016; Hollanders et al., 2017). Therefore, the current study assessed the function of ROCK signaling in diabetes-induced hepatic inflammation, using db/db mice. Our study showed that CPT (0.25 or 0.5 g/kg), metformin (20 mg/kg), and fasudil (5 mg/kg) effectively downregulated Rho, ROCK1, and ROCK2 and suppressed ROCK signaling. Similar findings were obtained in HepG2 cells administered palmitic acid. Above findings indicated CPT alleviated diabetes-induced hepatic inflammation *via* Rho-kinase signaling.

NF-κB signaling, downstream of ROCK, has a critical function in controlling the inflammatory process in T2DM and is activated after IκBα phosphorylation (Xiang et al., 2002). Wide range releases of cytokines such as IL-6 TNF-α activate NF-κB, giving rise to the hypothesis that T2DM is an inflammatory condition. In addition, activation of NF-κB pathways conducts macrophage-induced lipolysis and develops progression to fast hyperglycemia and postprandial hyperglycemia (Dongsheng et al., 2005). Previous studies revealed Rho/ROCK/NF-κB signaling participates in inflammatory progression in experimental diabetes (Qin et al., 2010). Hence, we assessed effect of CPT on NF-κB pathways in db/db mice liver and palmitic acid–treated HepG2 cells. Our data demonstrated that promoted IKKβ, NF-κB phosphorylation, and greater IκBα degradation were observed in the pathological liver and HepG2 cells. CPT (0.25 or 0.5 g/kg) remarkably suppressed Rho/ROCK/NF-κB signaling in diabetic animals, confirming that CPT successfully reduces the expression levels of ROCK, with a potential inhibitory role in NF-κB activation. These results suggested that CPT effectively inhibited diabetic-induced hepatic inflammation *via* Rho/ROCK/NF-κB signaling pathways.

Overall, we successfully identified the function of Rho-kinase signaling in diabetes-associated hepatic inflammation and illustrated the underlying mechanisms of Rho/ROCK/NF-κB-mediated inflammation. CPT was shown to have protective effects on the liver, using the db/db mouse model as well as the HepG2 cell line, and might improve diabetes-induced liver inflammation *via* ROCK signaling. CPT with reduced toxicity and fewer undesirable effects exerts a beneficial adjuvant drug for diabetes and its complications.

## Ethics Statement

All animal experimental procedures had approval from the Animal Ethics Committees of China Pharmaceutical University (CPU.2012-003) and were performed according to the National Institutes of Health Guidelines for the Care and Use of Laboratory Animals (NIH Publications No. 8023, revised 1978).

## Author Contributions

CJ and YW performed the experiments and wrote the manuscript. QJ, DZ, MG, and NY contributed to sample collection and data analysis. ZY, JZ, and SM designed the research study. All authors participated in manuscript revision, and approved the final version prior to submission.

## Funding

The current study was funded in part by the National Natural Science Foundation of China (Nos. 81503316 and 81872984), the Natural Science Foundation of Jiangsu Province (No. BK20161460), and Traditional Chinese Medicine Science and Technology Project of Jiangsu Province (Nos. YB2017031 and YB2017028).

## Conflict of Interest Statement

The authors declare that the research was conducted in the absence of any commercial or financial relationships that could be construed as a potential conflict of interest.

## Abbreviations

CP, *Cyclocarya paliurus* (Batal.) Iljinskaja (*C. paliurus*); CPT, *Cyclocarya paliurus* (Batal.) Iljinskaja (*C. paliurus*) triterpene fraction; OGTT, oral glucose tolerance test; AST, aspartate amino-transaminase; ALT, alanine aminotransferase; TG, triglyceride; TC, total cholesterol; IL-1β, interleukin-1β; IL-6, interleukin-6; TNF-α, tumor necrosis factor-α; ELISA, enzyme-linked immunosorbent assay; DMEM, Dulbecco’s modified eagle’s medium; FBS, fetal bovine serum; DMSO, dimethyl sulfoxide; SDS-PAGE, SDS-polyacrylamide; PVDF, polyvinylidene fluoride; ANOVA, one-way analysis of variance; IKKs, IκB kinases.

## Supplementary Material

Figure S1 CPT’s effects on the Rho-kinase pathway in palmitic acid–treated LO2 cells. Data are mean ± SD. ^##^p<0.01, ^#^P<0.05 vs. control cells. **p<0.01, *p<0.05 vs. palmitic acid group.

Figure S2 Graphical abstract of the manuscript.
