# Supplementary figures and images for: Cyclocarya paliurus Triterpenoids Improve Diabetes-Induced Hepatic Inflammation via the Rho-Kinase-Dependent Pathway
Source: Front Pharmacol. 2019 Jul 25;10:811. doi: 10.3389/fphar.2019.00811 (PMC6669819; doi:10.3389/fphar.2019.00811)

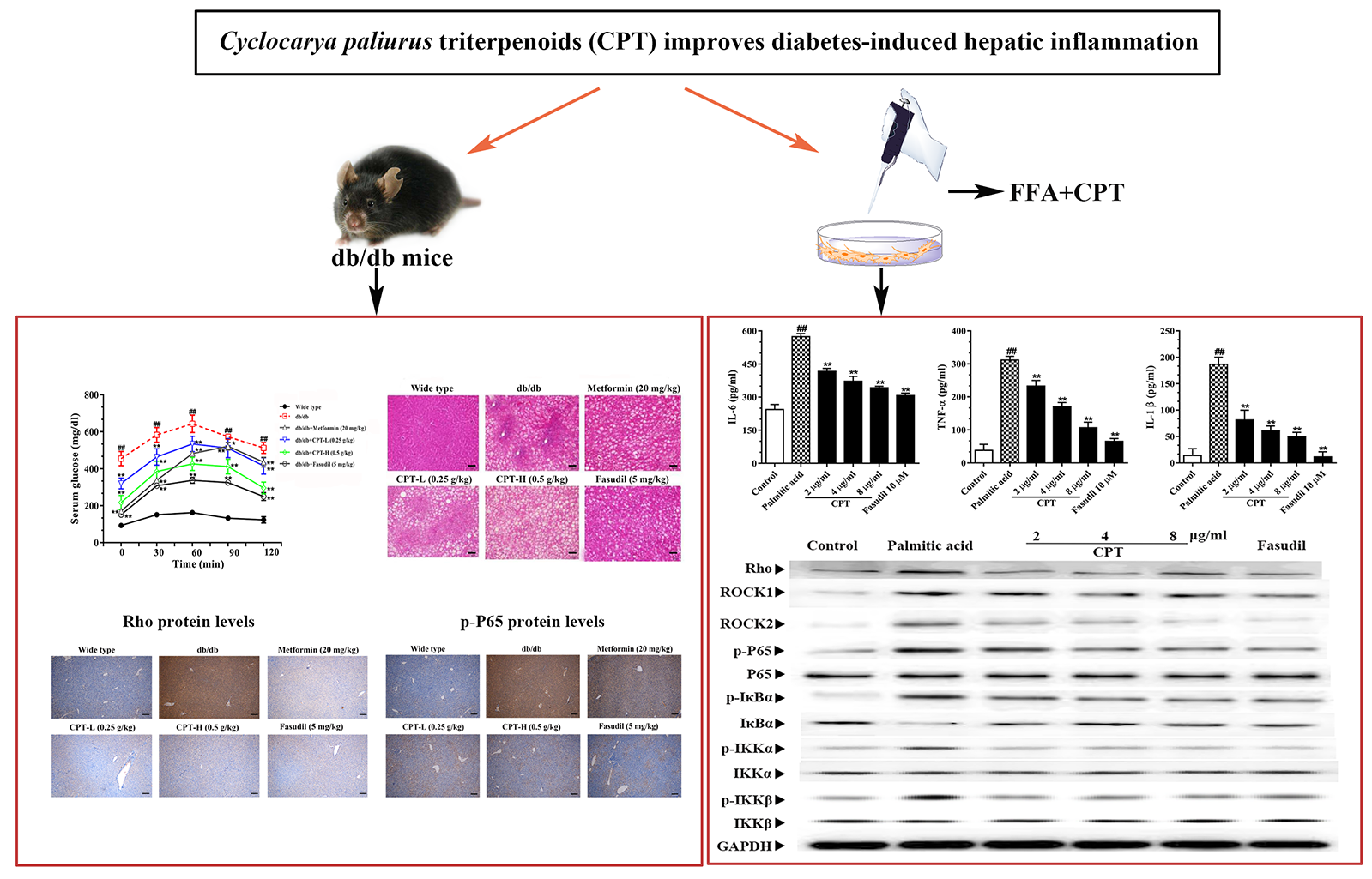

Supplement: Figure S1 — CPT’s effects on the Rho-kinase pathway in palmitic acid–treated LO2 cells. Data are mean ± SD. ##p<0.01, #P<0.05 vs. control cells. **p<0.01, *p<0.05 vs. palmitic acid group. [file Image_1.tif]

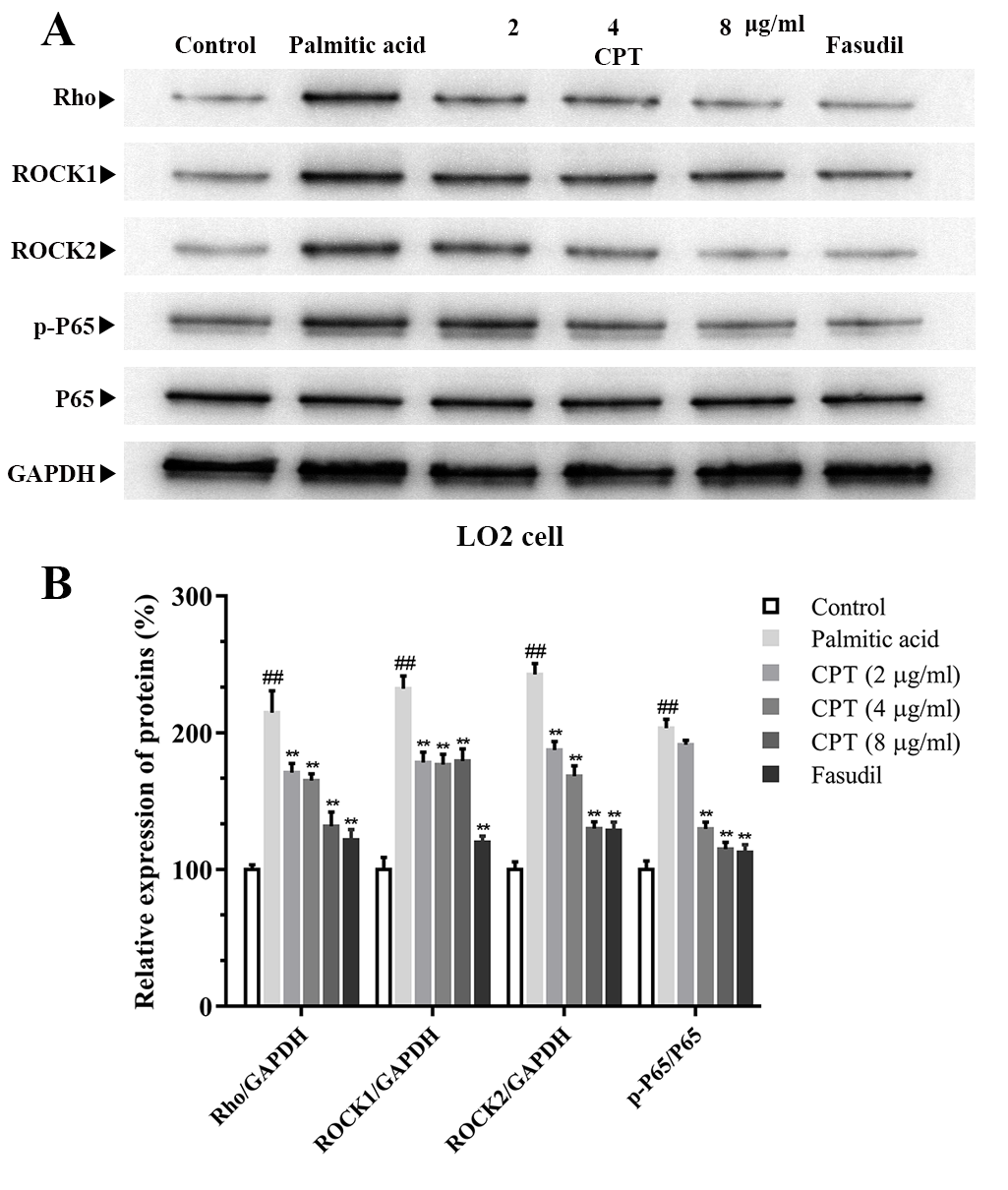

Supplement: Figure S2 — Graphical abstract of the manuscript. [file Image_2.tif]
